# Aspalathin-Rich Rooibos Tea Extract Regulates Hepatic Lipid Metabolism and Gut Microbiota in High-Fat Diet Fed Mice

**DOI:** 10.3390/nu18152452

**Published:** 2026-07-27

**Authors:** Xing Li, Juan He, Khalid S. Ibrahim, Michal R. Baran, James Reilly, Christo J. F. Muller, Johan Louw, Hui-Rong Jiang, Xinhua Shu

**Affiliations:** 1 Center for Biomedical Innovation and Technology, Pu Ai Medical School, Shaoyang University, Shaoyang 422000, China; lixing3971@hnsyu.edu.cn; 2 Medical College, Hunan University of Medicine, Huaihua 418000, China; hijl_0230@163.com; 3 Department of Biology, College of Science, University of Zakho, Zakho 42002, Kurdistan Region, Iraq; khalid.ibrahim@uoz.edu.krd; 4 Department of Biological and Biomedical Sciences, Research Centre for Health, School of Health and Life Sciences, Glasgow Caledonian University, Glasgow G4 0BA, UK; michal.baran@gcu.ac.uk (M.R.B.); j.reilly@gcu.ac.uk (J.R.); 5 Diabetes Discovery Platform, South African Medical Research Council, Cape Town 7505, South Africa; christo.muller@mrc.ac.za (C.J.F.M.); johan.louw@mrc.ac.za (J.L.); 6 Strathclyde Institute of Pharmacy & Biomedical Sciences, University of Strathclyde, Glasgow G4 0RE, UK

**Keywords:** aspalathin, rooibos tea, lipid metabolism, triglycerides, cholesterol, gut microbiota

## Abstract

**Background:** Hepatic lipid metabolism disorder has been linked to a wide range of diseases. Aspalathin is a flavonoid enriched in rooibos tea and has shown capacity to protect against hyperlipidemia, oxidative stress and inflammation. In the current study, we investigated the functional role of aspalathin in regulating liver metabolism and gut microbiota in the context of a high-fat diet (HFD). **Methods:** Male mice were fed a control diet or HFD, then HFD-fed animals were treated with or without aspalathin-rich extract (ASE). Hepatic neutral lipids were detected using oil red O staining and a colorimetric assay. Expression of cholesterol metabolism-, lipogenesis- and fatty acid β-oxidation-associated genes was measured. Gut microbiota was analyzed using 16S rRNA sequencing and bioinformatic approaches. **Results:** HFD-fed mice had markedly higher levels of triglycerides and cholesteryl esters and downregulated expression of cholesterol metabolism-, transport- and lipogenesis-related genes in the liver. ASE administration counteracted HFD-induced effects. In addition, HFD altered the composition, diversity and metabolic pathways of the gut microbiota. The richness of beneficial bacteria, particularly the butyrate-producing bacteria, was significantly decreased. The altered metabolic pathways are involved in the metabolism of carbohydrates, lipids, amino acids and nucleotides, as well as mitochondrial function. ASE treatment reversed most of the alterations back to the characteristics of the control group. **Conclusions:** Our results demonstrated the potential of ASE improving liver lipid metabolism and gut microbiota in the scenario of a high-fat diet, providing insights into future studies on the mechanism of ASE regulating lipid metabolism disorder.

## 1. Introduction

Lipid metabolism is a finely regulated complex biochemical reaction that involves digestion, absorption, synthesis, storage, utilization and degradation of body fat with the help of multiple related enzymes and is essential for cellular homeostasis and overall health [1,2]. Disruptions in lipid metabolism increase the risk of developing various diseases, including hyperlipidemia, hypercholesterolemia, obesity, non-alcoholic fatty liver disease, cardiovascular disease, coronary heart disease and cancer [3]. These diseases have become serious challenges for global public health. Modern dietary patterns (for instance, the Western diet), sedentary lifestyles and environmental stressors promote the prevalence of lipid metabolism disorders [4]. Although there are many drugs available on the market that are used for modulating lipid metabolism disorders, such as cholesterol/lipid-lowering synthetic drugs, they can have adverse effects. There is a need to develop modulators for lipid metabolism disorders with minimal or no side effects from natural plants.

The gut and liver play crucial roles in lipid metabolism, with their bidirectional communication, known as the gut–liver axis, that is regulated by bile acids, gut microbiota and their metabolites [5]. The gut is responsible for the digestion and uptake of dietary fats, which occurs primarily in the upper small intestine [6]. The liver is considered the primary site for lipid synthesis and transport that plays a critical role in lipid homeostasis [7]. Recently, the impact of gut microbiota on lipid metabolism and host health has received considerable attention. The gut microbiota, as a large group of complex commensal microorganisms within the human body, has been closely linked to host nutrient absorption, metabolic regulation, and immune responses [8,9]. Accumulated evidence suggests that gut microbiota serves as a vital regulator of host lipid homeostasis through regulating intestinal barrier function, energy and metabolic homeostasis, and affecting amino acid metabolism and the production of short-chain fatty acids [1,4,10]. In recent years, a diversity of plant extracts including *Eucommia* bark/leaf extract [11], *Arctium lappa* L. root extract [12], red yeast rice extract [13] and persimmon leaf extract [14], have been demonstrated to improve host lipid metabolism disorders by regulating the gut microbiota and its metabolites. The above findings highlight the potential value of plant extracts in improving lipid metabolism disorders via the regulation of intestinal microflora and host lipid metabolism.

Rooibos tea *Aspalathus linearis* (Burm.f.) R. Dahlgren is a woody shrub belonging to the genus *Aspalathus* from the *Fabaceae* family, which grows natively in the mountainous area of the western Cape Province of South Africa [15]. Its needle-like leaves and fine stems are commonly used for producing unfermented and fermented herbal teas. Rooibos tea has a long history of traditional use for relieving allergies, asthma, infantile colic, dermatological problems, and other gastrointestinal complaints, for example nausea and heartburn [16,17]. Previous studies have shown that rooibos tea and its extract possess hypoglycemic [18,19], cardioprotective [20,21], neuroprotective [22] and antitumor [23,24] effects, which are mainly attributed to its active components exerting α-glucosidase inhibitory, anti-inflammatory and anti-oxidative stress activities. Its major bioactive compound, known as aspalathin (2′,3,4,4′,6′-pentahydroxy-3′-C-*β*-D-glucopyranosyl dihydrochalcone), is a natural *C*-linked glucosyl dihydrochalcone that is highly abundant in unfermented rooibos aqueous extract [15]. Recently, it has been reported that three different rooibos preparations have demonstrated potential prebiotic-like functions on several probiotic organisms [25]. In support of this, a beneficial prebiotic-like effect of unfermented (green) rooibos tea and aspalathin has also been documented [26,27]. Although emerging evidence suggests that rooibos tea and aspalathin have various biological activities and pharmacological functions, studies of their effects on lipid metabolism and gut microflora are still lacking. In the present study, the influence of aspalathin-enriched rooibos tea extract on liver lipid metabolism and gut microbiota in the context of a high-fat diet is explored.

## 2. Materials and Methods

### 2.1. Animal Experiment

Aspalathin-rich extract (ASE) from unfermented rooibos tea contained 12.8% aspalathin (Product code: CPE-03287 and Batch Number: 730330), provided by Afriplex Pharmaceuticals PTY Ltd. (Paarl, South Africa). Quantification of aspalathin and other phenolic compounds presented in ASE by high-performance liquid chromatography diode array detection (HPLC-DAD) has been previously reported by Patel et al. [15]. All animal experimental procedures were approved by the Animal Welfare Ethics Committee of Shaoyang University (Ethical Approval No. 2020KJKT086, approval date 13 March 2020) and performed strictly following the institutional guidelines for the care and use of laboratory animals. Male C57BL/6J mice (6 weeks old, 18–22 g bodyweight) were obtained from Hunan Silaikejingda Experimental Animal Co., Ltd. (Changsha, China). Animals were maintained in the Animal Unit of Shaoyang University with a 12 h light/12 h dark cycle and free access to food and water. Following one week of acclimatization, animals were randomly divided into three groups (10 per group), designated as the control normal diet (CND) group; the high-fat diet group (HFD); and the high-fat diet and treatment with ASE group (HFD + ASE). Animals were maintained with normal chow diet (water ≤ 10%, total protein ≥ 18%, total fat ≥ 4%, total fiber ≤ 5%, total ash ≤ 8%, lysine ≥ 0.82%, methionine + cystine ≥ 0.53%) or high-fat diet (consisted of 70.8% basal diet, 10% egg yolk, 10% lard, 1% cholesterol, 8% sucrose, 0.2% bile salt) for 15 weeks. For the HFD + ASE group, the mice received ASE aqueous solution (3 mg/mL) via oral gavage at a dose of 40 mg/kg body weight, guided by previous publications [28,29,30]. Administration of ASE took place every two days for 32 days. To avoid possible degradation, the ASE solution was freshly prepared by reconstituting ASE powder in saline prior to administration. Mice in the CND and HFD groups were given saline solutions at the same volume via the intragastric route. At the end of the treatment, animals were anesthetized with 1% sodium pentobarbital through intraperitoneal injection at a dose of 50 mg/kg, followed by cervical dislocation. The cecum segments and liver samples were immediately collected under sterile conditions. The segments were subsequently opened along their cephalocaudal axis using sterile scissors, and the cecal contents were thoroughly harvested using a spatula. The acquired cecal contents and liver samples were stored at −80 °C until analysis.

### 2.2. Measurement of Triglycerides and Cholesterol in Mouse Livers

Mouse liver samples were immersed in hexane: isopropanol (3:2) and sonicated for 10 s, then incubated at room temperature for 30 min. The extract was centrifuged at 100× *g* for 10 min. The organic phase was transferred into a new microtube and air-dried at 50 °C. The dry lipids were resuspended in the assay buffer; the total triglycerides and cholesterol were measured using EnzyChrom Triglyceride assay kit (BioAssay system, UK) and Amplex Red Cholesterol Assay kit (Thermo Fisher Scientific Inc., Paisley, UK), respectively, following the manufacturer’s protocols.

### 2.3. Hematoxylin and Eosin (H-E) Staining

Liver samples were fixed with 4% paraformaldehyde (PFA)/PBS for 48 h, then dehydrated and incubated with a series of ethanol (10%, 30%, 50%, 70%, 90%, 100% ×3 ethanol each for 1 h), followed by two changes in histo-Clear, then embedded in paraffin and cut into 8 µm sections. The sections were subjected to deparaffinization and hydration, then hematoxylin and eosin staining following a standard protocol.

### 2.4. Oil Red O Staining

The liver samples were fixed in 4% phosphate-buffered formalin acetate at 4 °C overnight and then embedded with optimum cutting temperature (OCT) compound. The embedded samples were frozen at −20 °C for 30 min, then cut into 8 µm sections in a freezing microtome (PSZ-3550, Shenyang, China). The sections were mounted on SuperFrost glass slides and incubated with oil red O working solution (1.6 mg/mL in 60% isopropanol) for 15 min, then dipped once in 60% isopropanol and once in deionized water. The sections were then co-stained with Mayer’s Hematoxylin (Cat. No. MHS80, Sigma, Dorset, UK) for 3 min, dipped in deionized water 10 times, and mounted with Vectamount Aqueous Mounting Medium (Cat. No. H-5501, Vector Labs). Images were captured using an Olympus microscope. Quantification of oil red O signals was performed using Image J software version 1 (https://imagej.net/ij/, accessed on 20 January 2026). Each image was split into green, blue and red channels, then the red channel signal was quantified. The average of the control signal was used to convert the data into a percentage.

### 2.5. Quantitative Real-Time Polymerase Chain Reaction (qRT-PCR) Analysis

Total RNA extraction from the liver was performed with Trizol reagent (Catalog No. 15596026, Thermo Fisher Scientific Inc., Paisley, UK) following the manufacturer’s guidance. The RNA was reverse transcribed to cDNA using a reverse transcription kit (CW2569, Cwbio, Beijing, China). qRT-PCR was carried out using the PikoReal 96 Real-Time PCR System (Thermo Fisher Scientific Inc., Paisley, UK), along with UltraSYBR Mixture (CW2601, Cwbio, Beijing, China). Relative mRNA expression of candidate genes was calculated by the comparative CT method (2^−∆∆CT^) using *β-actin* as the reference gene. The sequences of gene-specific primers are listed in Table S1.

### 2.6. Western Blot

The amount of 0.25 g liver tissue was weighed, washed with ice-cold PBS, cut into small pieces, and placed in 300 μL ice-cold RIPA lysis buffer. Homogenization was performed in 1.5 mL EP tubes with the homogenizer (BioPrep-24, Hangzhou, China) at 4 °C. After homogenization, the EP tubes were placed on ice for 10 min. The homogenate was centrifuged at 12,000 rpm for 15 min at 4 °C. The supernatant was transferred to a clean 1.5 mL EP tube. The protein concentration was measured using the Bradford method. For immunoblotting, SDS-PAGE gels were prepared and loaded with 20 µg of protein per well. Following the electrophoresis, proteins were transferred to a nitrocellulose membrane. The nitrocellulose membranes were blocked with 5% semi-skimmed milk powder in Tris-buffered saline with Triton-X-100 for 1.5 h at room temperature, while shaking. The antibodies of ACC1 (1:1000, AWA10389), FASN (1:1000, AWA63697), CPT1 (1:1000, AWA04371), PPARα (1:500, AWA43094), SREBP-1c (1:500, AWA11276) were obtained from Changsha Abiowell Biotechnology Co., Ltd. (Changsha, China) and their respective anti-mouse HRP and anti-rabbit HRP conjugated antibodies (1:5000) were used for Western blotting. The β-actin (66009-1-Ig, Proteintech, Manchester, UK) antibody was used as an internal control. HRP-bound proteins were detected with ECL Plus Western blotting substrate (AWB0005, Changsha Abiowell, Changsha, China) using a ChemiScope6100 device (Clinx, Shanghai, China).

### 2.7. 16S rRNA Sequencing and Processing

Based on our experience in analyzing microbiota data, six animals per group will yield sufficient data to achieve biologically and statistically significant results. DNA was extracted from mouse cecal contents (7 samples from the CND group, 6 samples from the HFD group, and 6 samples from the HFD + ASE group) using the soil DNA extraction Kit (Qiagen, Manchester, UK). Following DNA extraction and purification, a library was constructed for subsequent second-generation sequencing of 16S rRNA (V3 + V4, 338F: 5′-ACTCCTACGGGAGGCAGCA-3′, 806R: 5′-GGACTACHVGGGTWTCTAAT-3′) on Illumina HiSeq 2500. To obtain high-quality sequences with tags, the raw sequencing reads were merged using FLASH (version 1.2.11) [31], and quality-filtered by Trimmomatic (version 0.33) [32], followed by chimera removal using UCHIME (version 4.2) [33]. The high-quality sequences with at least 97% identity were used to generate operational taxonomic units (OTUs) using USEARCH (version 10.0) [34]. OTUs were filtered out with a threshold of 0.005% of the total sequences [35]. Annotation and taxonomical analysis of representative sequences of OTUs were performed by searching against the Genome Taxonomy Database (GTDB, https://gtdb.ecogenomic.org/, accessed on 14 July 2026) and using the Ribosomal Database Project (RDP) Classifier (version 2.2) [36]. The relative abundance of each OTU was determined by counting the number of tags in each sample. Community composition was classified at the phylum and genus levels using the QIIME 2 software (version 2020.8) [37]. The MetaCyc and Kyoto Encyclopedia of Genes and Genomes (KEGG) Ortholog pathways of gut microbiota in the three groups were determined using the QIIME 2 software (version 2020.8).

### 2.8. Statistical Analysis

Significance of differences was analyzed using one-way analysis of variance (ANOVA), followed by Tukey’s or Bonferroni’s post hoc test where appropriate, using GraphPad Prism (version 10.3.0) or R software (version 4.5.2). The data are presented as mean ± SEM. The value of *p* < 0.05 was considered significant.

## 3. Results

### 3.1. ASE Alleviated Dysregulation of Hepatic Lipid Metabolism in HFD-Fed Mice

**Figure 1:**
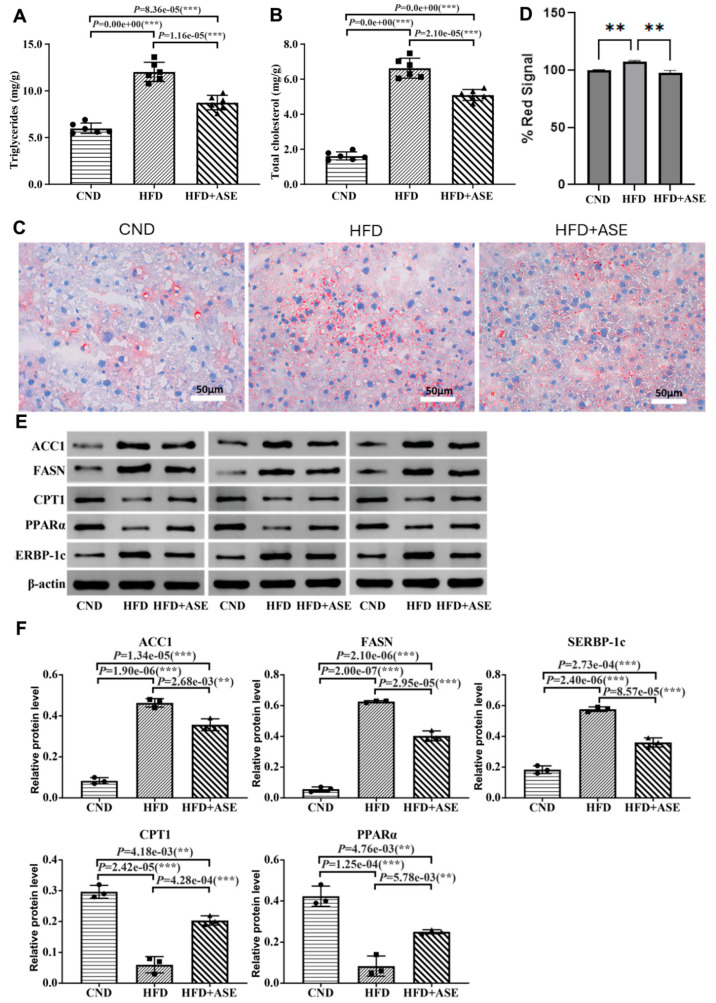
Effects of aspalathin-rich extract (ASE) on hepatic lipid metabolism. (**A**) Levels of liver triglycerides in CND, HFD, and HFD + ASE mice. The content expressed as units of mg per g fresh liver tissue (*n* = 6). (**B**) Liver total cholesterol of CND, HFD, and HFD + ASE mice. The content is expressed as units of mg per g fresh liver tissue (*n* = 6). (**C**) Oil red O staining for detection of neutral lipid accumulation in the liver samples of the control, HFD and HFD + ASE groups. (**D**) Quantitative analysis of oil red O (ORO) staining signals. (**E**) Immunoblotting analysis of triglyceride metabolism-related proteins in liver tissue. (**F**) Quantitative results of Western blot. The band intensity of individual targeted proteins was normalized to the band intensity of β-actin. Three individual samples of each group were used for Western blot. ** *p* < 0.01, *** *p* < 0.001. CND: control normal diet; HFD: high-fat diet; HFD + ASE: high-fat diet + aspalathin-rich extract.

Animals were allocated into three groups: one group was fed with CND, and two groups were fed with HFD. After 15 weeks of feeding, animals fed with HFD had significantly increased bodyweight compared to animals fed with CND; ASE-treated animals (HFD + ASE group) had slightly less bodyweight than that of the HFD group, though there was no significant difference (Figure S1). Initially, we examined hepatic histomorphology by hematoxylin and eosin (H&E) staining. The HFD mouse liver showed distinct morphological changes with vacuolation. ASE-treated mice demonstrated similar hepatic morphology to that of CDN mice (Figure S2). We then measured total triglycerides and cholesterol in the livers of untreated and ASE-treated animals using colorimetric assays. HFD-fed mice had significantly increased triglycerides and cholesterol in the liver, compared to mice that were fed a normal diet; ASE-treated animals had a marked decrease in triglycerides and cholesterol in the liver, compared to HFD-fed mice (Figure 1A,B). Furthermore, we used ORO staining to detect neutral lipids including triglycerides and cholesterol esters. The signals for ORO staining in HFD-fed mouse livers were significantly higher than those of the CND group (*p* = 0.0066, indicating HFD-induced accumulation of neutral lipids in livers), while ASE treatment significantly decreased these signals (*p* = 0.0018, Figure 1C,D). Additionally, we examined the mRNA levels of genes associated with de novo lipogenesis, fatty acid β-oxidation, and cholesterol metabolism in untreated and treated mouse livers. As shown in Figure S3A, the expression of genes implicated in de novo lipogenesis (*Acc1*, *Fasn* and *Srebp-1c*) in HFD group animals was significantly upregulated in comparison to the CND group, while ASE treatment rendered their expression significantly decreased. In contrast, the expression of fatty acid β-oxidation-related genes (*Cpt1* and *Pparα*) was markedly decreased in HFD mice; ASE treatment reversed the effects. Additionally, Western blot demonstrated that levels of ACC1, FASN and SERBP-1c were significantly increased, while levels of CPT1 and PPARα were significantly decreased, compared to the CND group; ASE administration significantly decreased the levels of ACC1, FASN and SERBP-1c, and increased the levels of CPT1 and PPARα (Figure 1E,F and Figure S4), compared to the HFD group. Expression of cholesterol transporters (*Abca1* and *Abcg1*), cholesterol metabolism enzymes (*Cyp27a1* and *Cyp46a1*), and cholesterol metabolism regulator (*Nr1h3* encoding LXRα) was markedly decreased in the livers of HFD-fed animals, compared to that of the CND group, whereas ASE administration significantly increased expression of these genes (Figure S3B).

### 3.2. ASE Altered the Composition and Diversity of Gut Microbiota

To investigate the effect of ASE on the gut microbiota, we used QIIME 2 software to analyze the bacterial taxonomic profiles of mouse cecum samples. Initially there were 1,521,774 sequence reads obtained from all samples. After quality control filtering, chimera filtering and merging paired-end reads, 1,239,704 non-chimeric good quality reads were retained for analysis of bacterial composition and diversity (Table S2).

**Figure 2:**
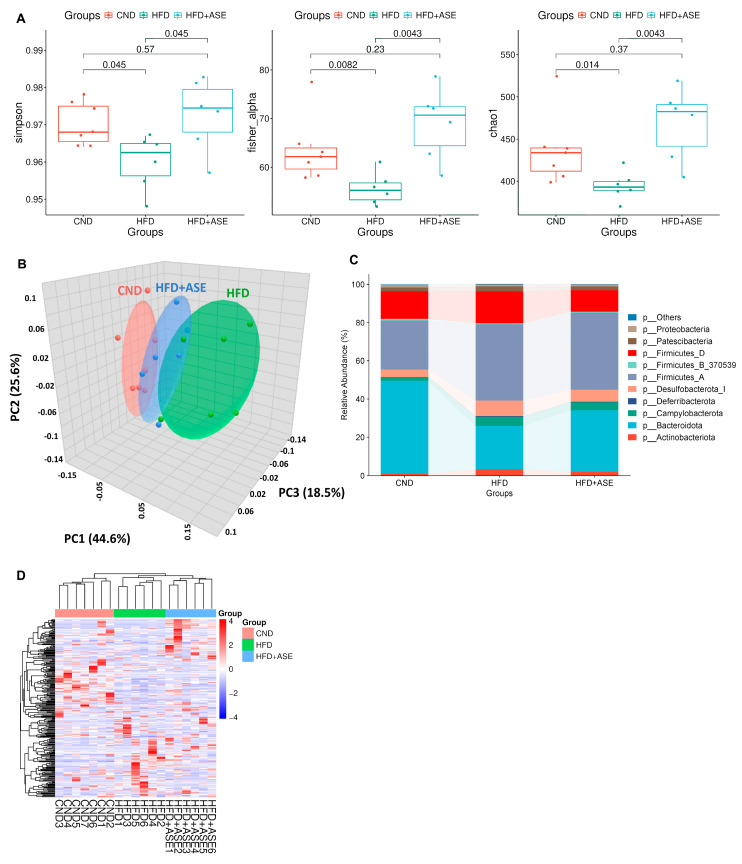
Effects of aspalathin-rich extract on the composition and diversity of mouse gut microbiota. (**A**) α diversity metrics (Simpson, Fisher_alpha and Chao 1) among the three animal groups. (**B**) β diversity (Jaccard index) among the three animal groups. (**C**) Relative abundance of the phyla in the three animal groups. (**D**) Heatmap representation of the phyla in the three animal groups. CND: control normal diet; HFD: high-fat diet; HFD + ASE: high-fat diet + aspalathin-rich extract.

Alpha (α) diversity measures bacterial diversity within individual samples, while beta (β) diversity measures the variation in the composition of bacterial communities between individual samples. We used α and β diversity to evaluate the microbiota diversity of the cecum samples from three experimental groups. Diversity metrics (Simpson’s index, Fisher_α index and Chao1 index) were significantly reduced in the HFD group compared to that of the CND group; the metrics in the HFD + ASE group were significantly increased compared to that of the HFD group, and in fact were close to or even higher than that of the CND group (Figure 2A). β diversity of the Jaccard index, which is used to quantify the dissimilarity between two groups, demonstrated a distinct separation of clustering of gut microbial structure between the CND and HFD groups, while the clustering of gut microbial structure in the HFD + ASE group was shifted, to some extent, closer to the CND group (Figure 2B).

To further investigate the differential abundance and phylogeny of taxa, a total of 240 taxa from 26 phyla were identified, consisting of 105 families and 150 genera (Table S3). The abundance and composition of gut microbiota varied among the three groups (Figure 2C). In the CND group, *Bacteroidota* was the most abundant phylum, followed by *Firmicutes*_A and *Firmicutes*_D; *Firmicutes*_A was the most abundant phylum in both HFD and HFD + ASE groups, followed by *Bacteroidota* and *Firmicutes*_D. The relative abundance of *Bacteroidota* in the HFD group was significantly less than that of both the CND and HFD + ASE groups. In contrast, the proportion of *Firmicutes*, *Desulfobacterota* and *Actinobacteriota* was significantly elevated in the HFD group compared to both the CND and HFD + ASE groups (Figure 2C). The heatmap analysis also showed that there were different clustering patterns among the three groups, indicating a clear separation of the gut microbiota profiles among these groups (Figure 2D).

**Figure 3:**
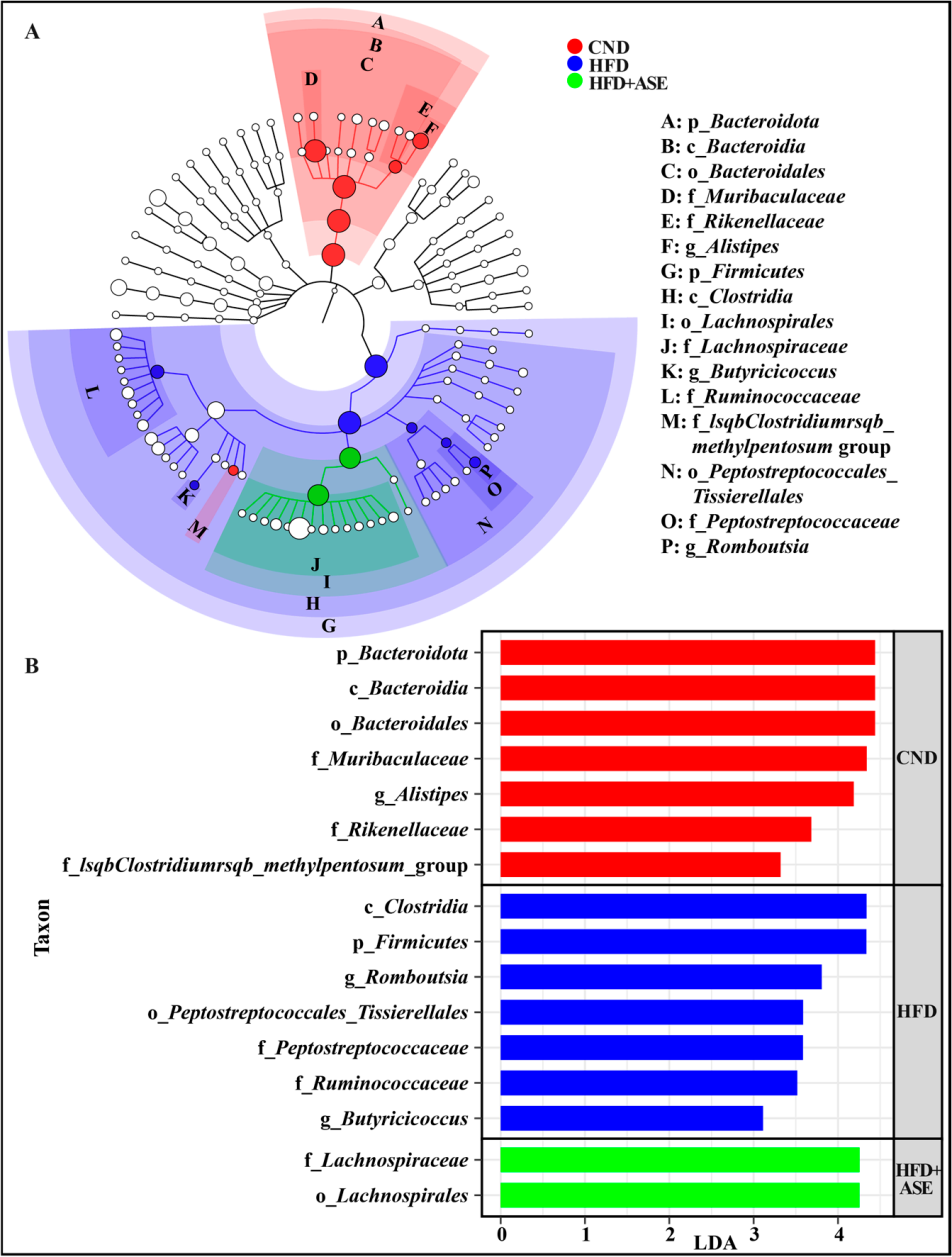
LEfSe analysis of bacterial taxa among three groups. (**A**) Cladogram of LefSe analysis results. (**B**) LDA discrimination column chart. LDA scores threshold = 3.0 and adjusted *p* < 0.05, Kruskal–Wallis tests with BH correction. The prefixes p, c, o, f, and g represented phylum, class, order, family and genus, respectively. CND: control normal diet; HFD: high-fat diet; HFD + ASE: high-fat diet+ aspalathin-rich extract.

Additionally, analysis of taxa differential abundance among the three groups was conducted using the LEfSe (LDA effect size) method with the standard of LDA > 3 and FDR-adjusted *p* < 0.05. As illustrated in the cladogram (Figure 3A), significantly differential features among the three groups (including 2 phyla, 2 classes, 3 orders, 6 families and 3 genera) were identified. At the family level, *Muribaculaceae*, *Rikenellaceae* and *lsqbClostridiumrsqb*_*methylpentosum*_groups were predominantly detected in the CND group; *Peptostreptococcaceae and Ruminococcaceae* were more abundant in the HFD group, while *Lachnospiraceae* was highly abundant in the HFD + ASE group. At the genus level, the bacteria from *Alistipes* were more abundant in the CND group. For the HFD group, *Romboutsia* and *Butyricicoccus* were the main elevated genera. These observations were also supported by LDA scores (Figure 3B), which further illustrated the microbial differences among the three groups. The *lsqbClostridiumrsqb*_*methylpentosum*_group family and members of the order *Bacteroidales* (*Muribaculaceae*, *Rikenellaceae*, *Alistipes*) with higher LDA scores were mainly related to the CND group. The bacteria with larger LDA scores from members of the order *Peptostreptococcales_Tissierellales* (*Peptostreptococcaceae*, *Romboutsia*) and *Oscillospirales* (*Ruminococcaceae*, *Butyricicoccus*) had a close relationship with the HFD group. However, taxa like *Lachnospirales* and *Lachnospiraceae* exhibited higher LDA scores, implying their potential functional role in the HFD + ASE group. Differential abundance analysis using the Songbird package also demonstrated similar results, as seen in Table S4. The members of the *Muribaculaceae* family with larger positive differential ranking values were more associated with the CND group, whereas the genera *Eubacterium siraeum* group and *IncertaeSedis* (belonging to the family *Ruminococcaceae*) with relatively negative differential ranking values were more correlated with the HFD group. For the HFD + ASE group, *Lachnospiraceae* bacteria had most features with positive differential ranking values and might therefore be potential biomarkers.

### 3.3. Aspalathin-Rich Extract Modulated Metabolic Pathways in the Gut Microbiota

**Figure 4:**
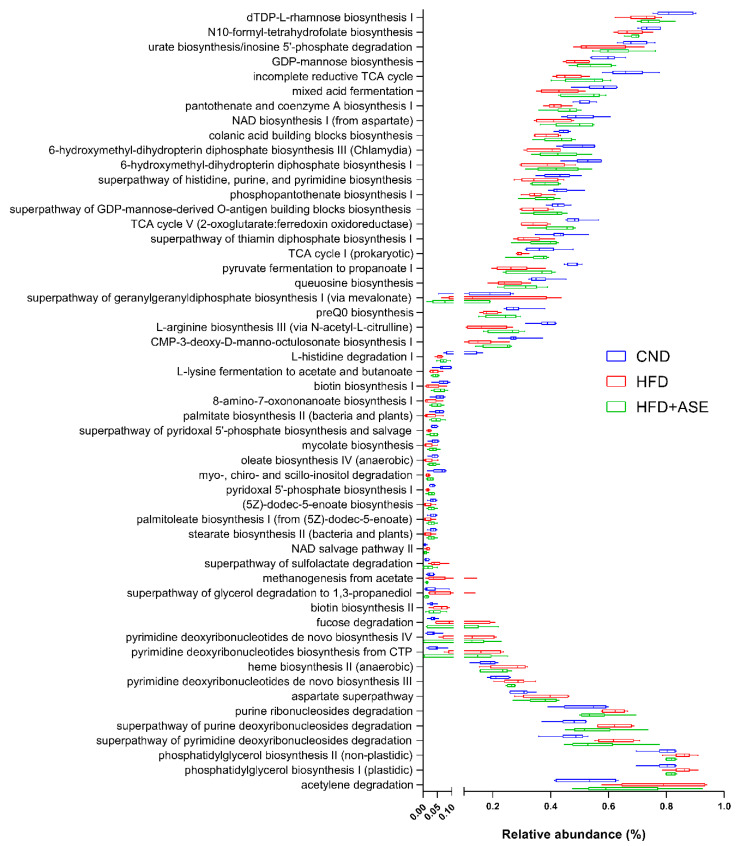
MetaCyc pathways lower or higher in the HFD group compared to those of both the CND and HFD + ASE groups. CND: control normal diet; HFD: high-fat diet; HFD + ASE: high-fat diet + aspalathin-rich extract.

To further elucidate the functional relevance of differentially abundant microbes, we conducted MetaCyc pathway functional annotation among the three groups using PICRUSt2. 396 metabolic pathways were predicted, and the abundance of 100 pathways was significantly changed among the three groups (Table S5). The abundance of 35 metabolic pathways—for example, pantothenate and coenzyme A biosynthesis I, biotin biosynthesis, and NAD biosynthesis I—in the HFD group was significantly lower than that of both the CND and HFD + ASE groups; 18 pathways—e.g., superpathway of purine deoxyribonucleoside degradation and phosphatidylglycerol biosynthesis—were enriched in the HFD group compared to that of both CND and HFD + ASE groups (Figure 4). The results demonstrate that HFD induced a significant functional shift in gut microbiota and that ASE administration fully or partially restored the functional pathways back to that of the CND group.

**Figure 5:**
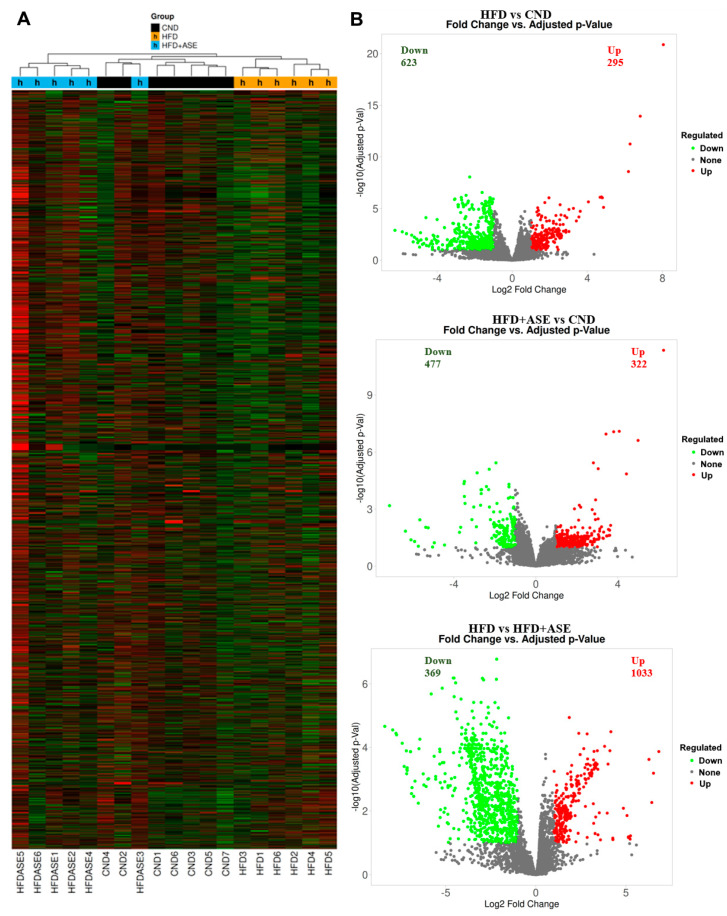
Aspalathin-rich extract modulated the abundance of Kyoto Encyclopedia of Genes and Genomes (KEGG) orthologs (KOs). (**A**) Heatmap of KO representation in individual samples among three groups. (**B**) Volcano plots demonstrate downregulated (labeled in green), upregulated (labeled in red), and unchanged (none, labeled in gray) KO identifiers, compared between the two groups. CND: control normal diet; HFD: high-fat diet; HFD + ASE: high-fat diet + aspalathin-rich extract.

**Figure 6:**
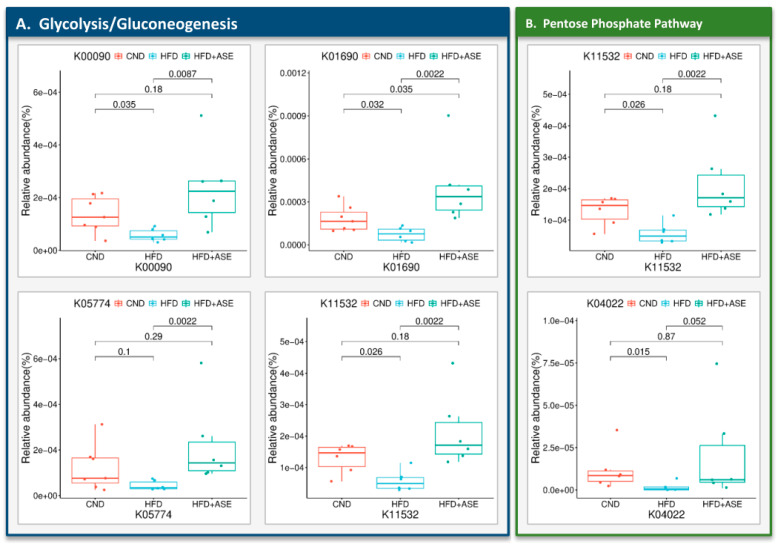
Representative differential KEGG ortholog pathways, which were involved in glycolusis/gluconeogenesis (K00090: glyoxylate/hydroxypyruvate/2-ketogluconate reductase; k01690: phosphogluconate dehydratase; k05774: ribose 1,5-bisphosphokinase; k11532: fructose-1,6-bisphosphatase II/sedoheptulose-1,7-bisphosphatase) and pentose phosphate pathway (k11532: fructose-1,6-bisphosphatase II/sedoheptulose-1,7-bisphosphatase; k04022: alcohol dehydrogenase). CND: control normal diet; HFD: high-fat diet; HFD + ASE: high-fat diet + aspalathin-rich extract. (**A**) Glycolysis/Gluconeogenesis; (**B**) Pentose Phosphate Pathway.

**Figure 7:**
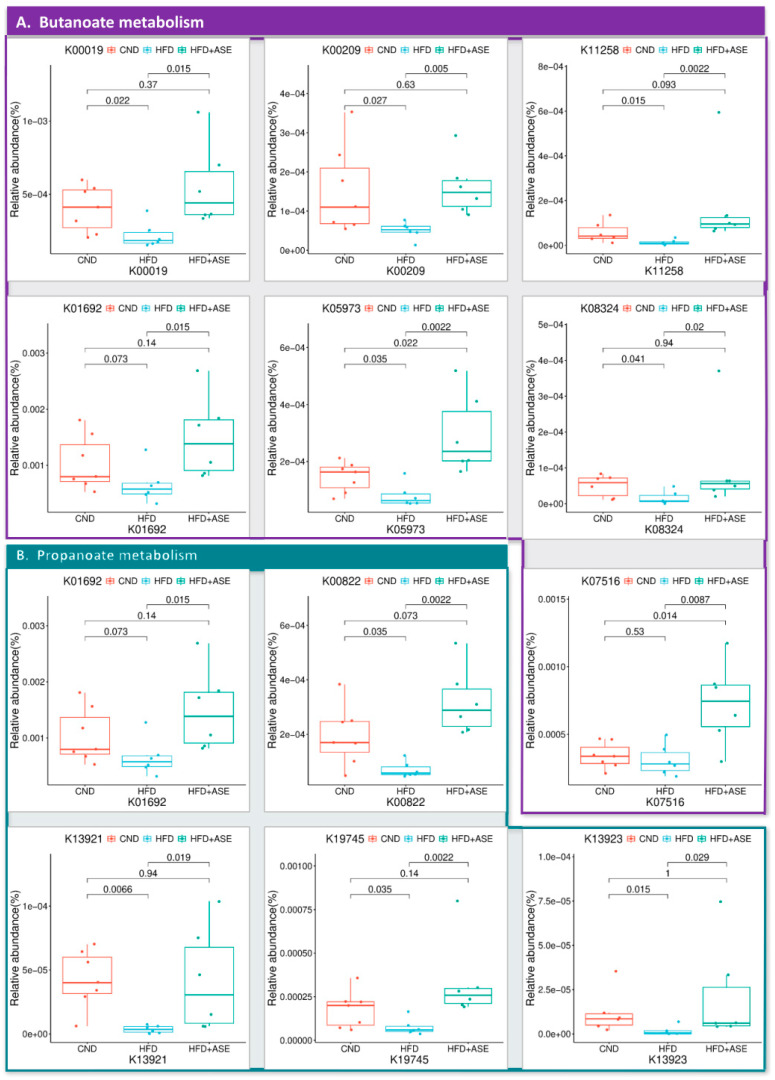
Representative differential KEGG ortholog pathways, which were involved in butanoate (k00019: 3-hydroxybutyrate dehydrogenase; k00209: enoyl-[acyl-carrier protein] reductase/trans-2-enoyl-CoA reductase; k11258: acetolactate synthase II small subunit; k01692: enoyl-CoA hydratase; k05973: poly(3-hydroxybutyrate) depolymerase; k08324: succinate-semialdehyde dehydrogenase; k07576: metallo-beta-lactamase family protein) and propanoate metabolism (k01692: enoyl-CoA hydratase; k00822: beta-alanine--pyruvate transaminase; k13921: 1-propanol dehydrogenase; k19745: acrylyl-CoA reductase; k13923: phosphate propanoyltransferase). CND: control normal diet; HFD: high-fat diet; HFD + ASE: high-fat diet + aspalathin-rich extract.

Additionally, in order to link the bacterial genes to their functional roles, we predicted 6489 pathways using the PICRUSt2 software. The heatmap and hierarchical clustering of these pathways demonstrated distinct clustering patterns with a clear separation of the HFD group from both the CND and the HFD + ASE groups (Figure 5A, Table S6). Further volcano plot analysis to compare differential abundance of functional KO identifiers between two groups based on the log2 fold changes and −log10 *p* value revealed that the abundance of 295 KO identifiers was significantly increased, and that of 623 KO identifiers significantly decreased, in the HFD group compared to the CND group; similarly, the activity of 322 KO identifiers was significantly increased, and that of 477 KO identifiers significantly decreased, in the HFD + ASE group compared to the CND group. The abundance of 1033 KO identifiers was significantly upregulated and that of 369 KO identifiers was significantly downregulated in the HFD group compared to the HFD + ASE group (Figure 5B). Among the significantly altered KO identifiers, functions of KOs involved in glycolysis/gluconeogenesis, including glyoxylate/hydroxypyruvate/2-ketogluconate reductase (k00090), phosphogluconate dehydratase (k01690), ribose 1,5-bisphosphokinase (k05774) and fructose-1,6-bisphosphatase II/sedoheptulose-1,7-bisphosphatase (k11532), were significantly decreased in the HFD group, compared to the CND group. ASE treatment significantly increased the activities of the pathway to similar levels as the CND group (Figure 6A). Functions of KOs associated with the pentose phosphate pathway including alcohol dehydrogenase (k04022) and fructose-1,6-bisphosphatase II/sedoheptulose-1,7-bisphosphatase (k11532) also presented a similar tendency in the three groups (Figure 6B). Activities of KOs involved in butanoate metabolism (k00019: 3-hydroxybutyrate dehydrogenase; k00209: enoyl-[acyl-carrier protein] reductase/trans-2-enoyl-CoA reductase; k11258: acetolactate synthase II small subunit; k01692: enoyl-CoA hydratase; k05973: poly(3-hydroxybutyrate) depolymerase; k08324: succinate-semialdehyde dehydrogenase; k07576: metallo-beta-lactamase family protein) and in propanoate metabolism (k01692: enoyl-CoA hydratase; k00822: beta-alanine--pyruvate transaminase; k13921: 1-propanol dehydrogenase; k19745: acrylyl-CoA reductase; k13923: phosphate propanoyltransferase) were significantly decreased in the HFD group, when compared to both the CND and HFD + ASE groups (Figure 7).

## 4. Discussion

ASE is rich in phenolic compounds, including dihydrochalcones (aspalathin and nothofagin), flavones (orientin, isoorientin, luteolin, luteolin-7-O-glucoside, vitexin and chrysoeriol), flavonols (rutin, hyperoside, querceti and isoquercitrin), phenylpropanoid (phenylpyruvic acid-2-O-glucoside, PPAG) and hydroxycinnamic acid (ferulic acid). Aspalathin is the predominant compound in ASE, accounting for about 13% of total compounds [15,38]. Previous studies indicate that ASE has multiple pharmacological activities, including free radical scavenging and antioxidant effects, anti-inflammatory properties, anti-diabetic action and protection against diabetes complications, anti-fatigue effects, cardiovascular protection, digestive system protection, and hepatoprotective effects [39]. It has been reported that ASE administration lowers the levels of total cholesterol, triglyceride, and LDL-C in the serum of normal and streptozotocin-induced diabetic rats [40,41]. Additionally, ASE decreased serum levels of total cholesterol and LDL in HFD-induced diabetic vervet monkeys [42]. To investigate the effects of ASE on liver lipid metabolism disorders, mice were fed with high-fat diet for 15 weeks to stimulate dysfunction in lipid metabolism and intervened with ASE for 32 days. The duration of high-fat diet feeding and treatment with ASE was determined by drawing parallels with previous studies of high-fat-induced obese or diabetic animal models [29,30,42,43], which exhibited lipid metabolism disorders. In these previous studies, disease models were treated with ASE for 4–6 weeks. For instance, Obasa et al. induced an obese Wistar rat model by feeding a high-fat diet for 16 weeks and treated the model with aspalathin-rich green rooibos extract (60 mg/kg/day) for 6 weeks [30]. Our results also supported and justified the choice of experimental duration. The results showed that the levels of neutral lipids (cholesteryl esters and triglycerides) were significantly increased in the liver of HFD mice, consistent with our previous study [44], and that ASE reversed HFD-induced effects. The accumulation of cholesterol storage in the HFD mouse liver was possibly caused by defects in cholesterol metabolism and transport, as the expression of cholesterol homeostasis-associated genes was significantly downregulated, and ASE administration upregulated the expression of these genes. ASE is proposed to inhibit fatty acid biosynthesis and enhance fatty acid β-oxidation [39]. Our results also demonstrated that ASE decreased the gene and protein expression involved in de novo lipogenesis and increased the gene and protein expression implicated in fatty acid β-oxidation. It would be worth investigating if the decreased triglyceride level in ASE-treated animals is due to activation of AMP-activated protein kinase (AMPK), the major regulator of fatty acid β-oxidation.

Diet plays a pivotal role in shaping the composition, diversity and function of the gut microbiota. HFD can induce dysbiosis of gut microbiota and is a primary risk factor for metabolic diseases, for example, obesity, diabetes and cardiovascular diseases [45]. Here, we also found that HFD-fed animals had significantly altered composition and reduced diversity of gut microbiota, and that ASE exposure partially reversed the changes back to levels similar to those of CND animals. Previous studies have shown that abundance of *Firmicutes* is increased, while abundance of *Bacteroidota* is reduced, in obese humans and rodents [45]. Here, we also found similar effects and observed that ASE treatment reversed these effects. The phylum *Bacteroidota* plays a critical role in liver lipid metabolism through the gut–liver axis. The interaction of *Bacteroidota* and the liver primarily occurs through the production of key metabolites. *Bacteroidota* can ferment dietary fiber to produce short chain fatty acids (SCFAs), e.g., propionate and acetate, which are transported into the liver, where they reduce de novo lipogenesis and enhance fatty acid oxidation by activating the AMPK signaling pathway. Specific species of *Bacteroidota* can modify primary bile acids to form secondary bile acids, which bind hepatic receptors to tightly regulate liver lipid homeostasis [46].

In addition, a signature taxon at the family level in the HFD + ASE group has been identified as *Lachnospiraceae*, which can produce butanoate by fermenting dietary polysaccharides. Interestingly, two studies have shown a significant increase in the relative abundance of *Dorea* (*Lachnospiraceae* family), *Lachnospiraceae* and *Lachnospiraceae*_NK4A136_group at the genus level in high-fat-induced obese rodents [47,48]. This implies a complex interplay between high-fat diet and gut microbiota. The gut microbial community imposes substantial influences on host lipid and glucose metabolism and energy homeostasis via altering the integrity of the intestinal barrier or their metabolites as signaling molecules. Previous studies indicated that 4 mmol/L butyrate enhanced the expression of occludin and ZO-1 genes in IPEC-J2 cells and elevated the expression of the claudin-1 gene in rat cdx2-IEC cells, decreased intestinal permeability and increased intestinal villus height in mice [49,50]. Moreover, butyrate can activate the AMPK pathway, promote fatty acid β-oxidation, and decrease fatty acid biosynthesis in the liver [51]. Furthermore, butanoate can lower cholesterol in the liver by inhibition of cholesterol biosynthesis and blockage of intestinal absorption [52,53]. The data indicated that ASE lowered the levels of cholesterol and triglycerides at least partially via modulation of butanoate-producing bacteria, though we did not measure the butanoate level in the cecum. Similarly, several studies have demonstrated that green tea extract [54] and other active phytochemicals such as ginsenoside Rd [55], tetrahydrocurcumin [56], resveratrol [57], and pseudolaric acid B [58] exert beneficial effects on metabolic disorders via regulating liver lipid metabolism and modulating gut microbiota, which are in line with our results.

The microbial MetaCyc pathway analysis demonstrated significant changes in many pathways among the three groups. These pathways are involved in multiple functions: for example, metabolism of lipids, amino acids, carbohydrates and nucleotides. Some metabolic pathways are particularly related to dysregulation of lipid metabolism described in the current study. The superpathway of geranylgeranyl diphosphate biosynthesis I (via mevalonate), which produces geranylgeranyl diphosphate (GGPP), was significantly enriched in the HFD group, compared to both the CND and HFD + ASE groups. GGPP is a crucial precursor for the biosynthesis of cholesterol. Therefore, enrichment of this superpathway is possibly associated with increased cholesterol in the liver of the HFD group. In addition, the tricarboxylic acid (TCA) cycle was significantly decreased in the HFD group, compared to both the CND and HFD + ASE groups. The TCA cycle is a central metabolic hub for oxidizing carbohydrates and fatty acids and the release of energy. Impaired flux of the TCA cycle can cause liver triglyceride accumulation [59]. Further analysis of KOs showed that many pathways were altered when a comparison was made between two groups. Some altered pathways induced by HFD were fully or partially reversed by ASE administration. Of particular interest, functions of KOs associated with glycolysis/gluconeogenesis, propanoate metabolism and butanoate metabolism were less abundant in the HFD group compared to the CND and HFD + ASE groups, consistent with analysis of MetaCyc pathways.

Aspalathin is the main phenolic compound in ASE; other phenolic compounds, including orientin, hyperoside, quercetin, vitexin, rutin, luteolin, PPAG, ferulic acid, and nothofagin, have been identified in ASE [15,38]. Aspalathin treatment has been shown to enhance lipid transport and metabolism and prevent lipid accumulation in H9c2 cells [60]. Orientin has been demonstrated to downregulate expression of lipogenesis genes and upregulate expression of lipolysis genes, resulting in decreased accumulation of intracellular triglyceride in mouse adipocyte 3T3-L1 cells [61]. Hyperoside can regulate cholesterol, bile acid and fatty acid metabolism in the liver and lower hepatic lipid levels in high-fat-diet-fed rats [62,63]. Quercetin also can regulate liver lipid metabolism via activating the AMPK-PPAR pathway, which enhances lipid β-oxidation and reduces fat deposition in rodents with non-alcoholic fatty liver disease [64]. Similarly, vitexin, rutin, luteolin and ferulic acid have been shown to modulate hepatic lipid metabolism [65,66,67,68]. Additionally, these individual phenolic compounds have been reported to regulate gut microbiota in rodents [63,64,66,67,68,69,70]. We showed that ASE regulated liver lipid metabolism and gut microbiota. It is possible that ASE’s effects depend on additive or synergistic actions of aspalathin and other flavonoids, which require further investigation.

Phenolic compounds can be metabolized by certain microbiota when reaching the colon, producing various metabolites. These metabolites can be taken up by intestinal epithelial cells and transported to peripheral and central organs, where they can participate in various biological functions [71]. Gut bacteria can metabolize aspalathin to small molecules, phenolic acids; similarly, gut bacteria can break down rutin to hyperoside, then to quercetin, which can further be metabolized by gut bacteria to simpler phenolic acids and active metabolites, such as hydroxyphenyllactic acid (HPLA). Vitexin can also be broken down by gut bacteria to various small phenols and aromatic acids [72]. Furthermore, gut bacteria can convert luteolin into smaller bioavailable metabolites like 3-(3,4-dihydroxyphenyl) propionic acid [73]. These gut bacterial-derived metabolites have been demonstrated to regulate lipid metabolism, including lowering cholesterol levels, suppressing lipogenesis, accelerating lipid oxidation, and reducing liver lipid accumulation [74]. It is reasonable that these metabolites may contribute to ASE’s capacity against liver lipid metabolic disorders, which warrants further studies.

There are several limitations in this study. Firstly, we found that the alteration of gut microbiota is supposed to result in changes in the production of SCFAs. However, we did not measure the levels of SCFAs in cecal contents or plasma in the current study. Future research will examine the levels of SCFAs in the cecum and in plasma of animals in the control, HFD and HFD + ASE groups; further, SCFAs mediate various signaling pathways in liver lipid metabolism, particularly the AMPK pathway, which requires further attention. Secondly, gut microbiota may also metabolize the main phenolic compounds of ASE into small molecules (metabolites), which can act as signal molecules in the regulation of liver lipid homeostasis. We will identify the metabolites from the main phenolic compounds of ASE in the cecum and in the plasma of ASE-treated animals. Thirdly, alterations in microbial functional pathways among the three groups were largely predicted; associated microbial metabolic activities require further comprehensive investigations.

## 5. Conclusions

In this study, we examined the effects of ASE on liver lipid metabolism and gut microbiota in the context of HFD. HFD elevated the levels of triglycerides and cholesterol in the liver and decreased the expression of cholesterol metabolism-, lipogenesis- and fatty acid β-oxidation-associated genes, and this was reversed by treatment with ASE. ASE could also partially reverse the changes in composition, diversity and metabolic pathways of gut microbiota caused by HFD. Alterations in gut microbiota may mediate ASE’s effects on regulation of hepatic lipid metabolism. The beneficial effects of ASE in regulating hepatic lipid metabolism and gut microbiota are possibly through additive or synergistic actions of aspalathin and other phenolic compounds. Our study provides preliminary evidence that ASE has therapeutic potential for lipid metabolism disorders.

## Data Availability

The 16S rRNA sequencing raw data presented in this study is available on the NCBI SAR (BioProject accession: PRJNA1461957).

## Supplementary Materials

The following supporting information can be downloaded at: https://www.mdpi.com/article/10.3390/nu18152452/s1. Figure S1: Bodyweight of animals at start point, before treatment and after treatment; Figure S2: Liver histology of animals from three groups; Figure S3: Quantitative analysis of triglycerides and cholesterol metabolism-associated genes in liver; Figure S4: Western blot raw images; Table S1: Sequences of primers used in qRT-PCR; Table S2: Reads data after preprocessing; Table S3: The identified phylogenetic of taxa; Table S4: Differential abundance analysis by songbird; Table S5: Functional annotation of MetaCyc pathways; Table S6: Functional annotation of KEGG orthology (KO) pathways.
