# Supplementary material for: Aspalathin-Rich Rooibos Tea Extract Regulates Hepatic Lipid Metabolism and Gut Microbiota in High-Fat Diet Fed Mice
Source: Nutrients. 2026 Jul 27;18(15):2452. doi: 10.3390/nu18152452 (PMC13468025; doi:10.3390/nu18152452)
Supplement: Supplementary file 1 [file nutrients-18-02452-s001.zip › nutrients-4422928-supplementary/Xing Aspalathin Supplementary Data.pdf]

Supplementary Material

List:

Table S1 Sequences of primers used in qRT-PCR.
Table S2 Reads data after preprocessing.
Table S3 The identified phylogenetic of taxa.
Table S4 Differential abundance analysis by songbird.
Table S5 Functional annotation of MetaCyc pathways.
Table S6 Functional annotation of KEGG orthology (KO) pathways.
NOTE: All supplementary tables are displayed in respective sheets in one separate excel file.

Figure S1 Bodyweight of animals at start point, before treatment and after treatment
Figure S2 Liver histology of animals from three groups.
Figure S3 Quantitative analysis of triglycerides and cholesterol metabolism-associated genes in liver.
Figure S4 Western blot raw images.

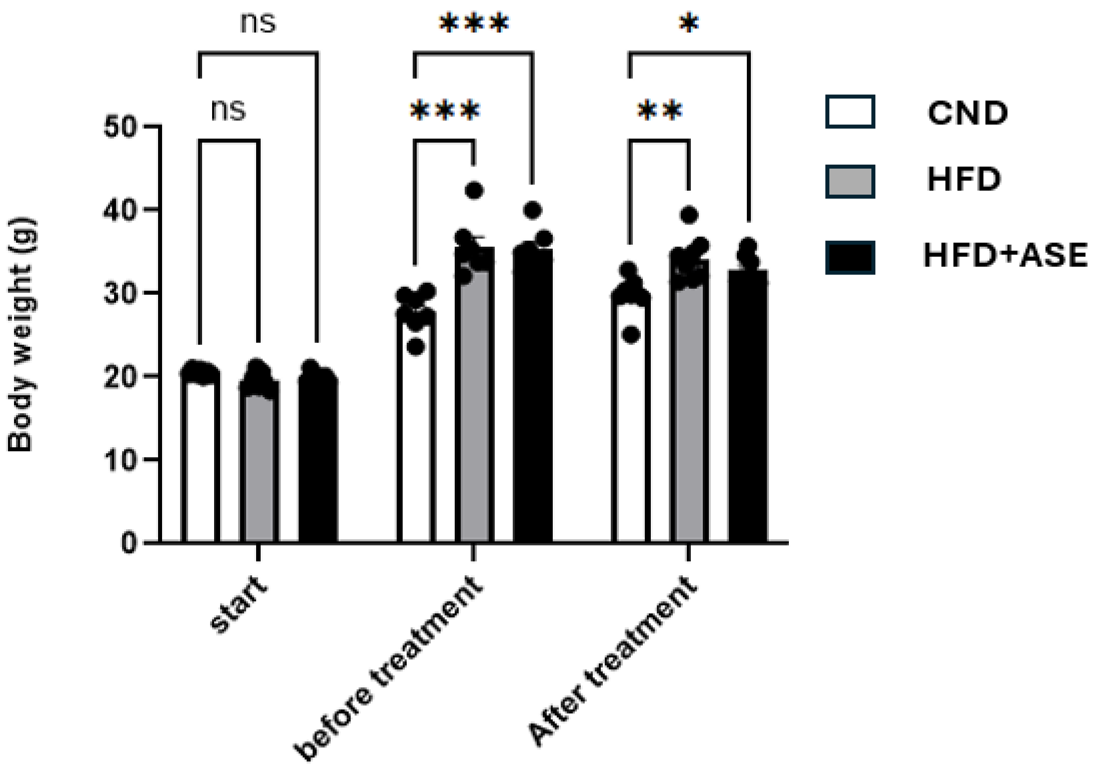

Figure S1 Bodyweight of animals at starting timepoint, before treatment timepoint and after treatment timepoint. The bodyweight was not different among three groups at the starting timepoint; the bodyweight of HFD and HFD+ASE groups were significantly higher than that of the CND group at the before-treatment timepoint; the bodyweight of HFD and HFD+ASE groups were significantly higher than that of the CND group at the after-treatment timepoint. $^{*}P < 0.05$, $^{**}P < 0.01$, $^{***}P < 0.001$. Bodyweight of both HFD and HFD+ASE groups were similar without significance. CND: control normal diet; HFD: high-fat-diet; HFD+ASE: high-fat-diet + aspalathin-rich extract.

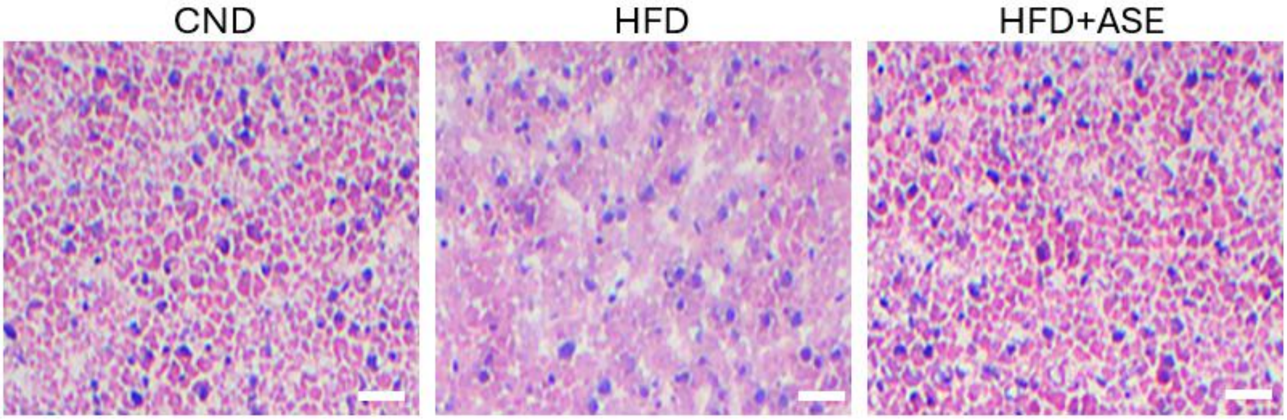

Figure S2 Liver histology of animals from three groups. The HFD animals showed clear morphological changes with vacuolation, compared to that of CND animals. The ASE treated animals had similar morphology to that of CND animals. CND: control normal diet; HFD: high-fat-diet; HFD+ASE: high-fat-diet + aspalathin-rich extract. Scale bar: 50µm.

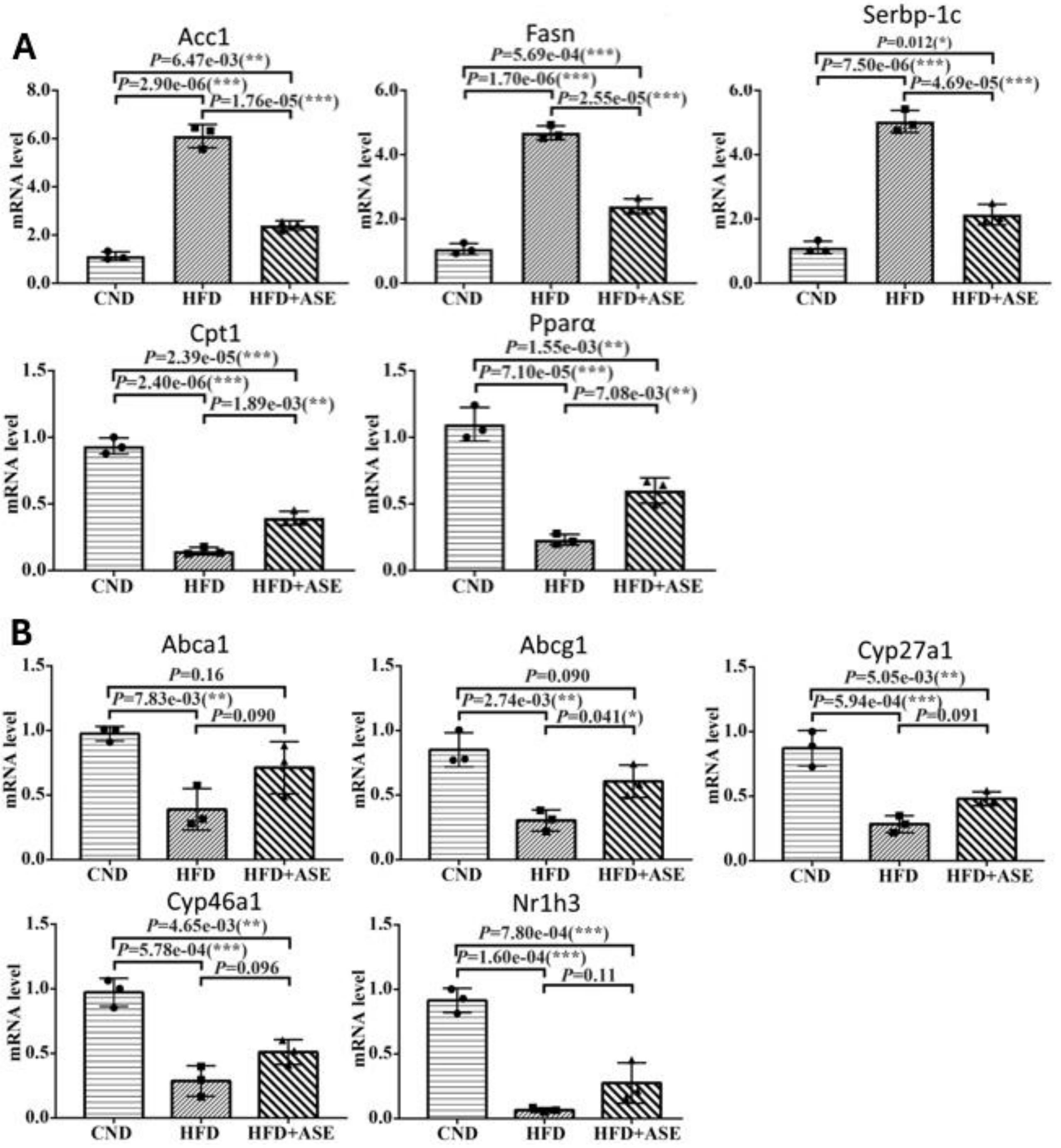

Figure S3 Effect of aspalathin-rich extract (ASE) on expression of triglycerides and cholesterol metabolism related genes in mouse liver samples, measured by qRT-PCR. (A) Expression of triglycerides metabolism related genes. (B) Expression of cholesterol metabolism related genes. Significance: *$P < 0.05$, **$P < 0.01$, ***$P < 0.001$. CND: control normal diet; HFD: high-fat-diet; HFD+ASE: high-fat-diet + aspalathin-rich extract.

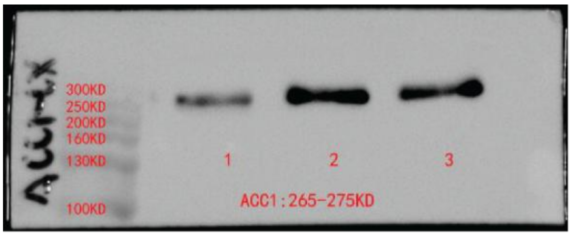
300KD
250KD
200KD
160KD
130KD
100KD
1 2 3
ACC1:265-275KD
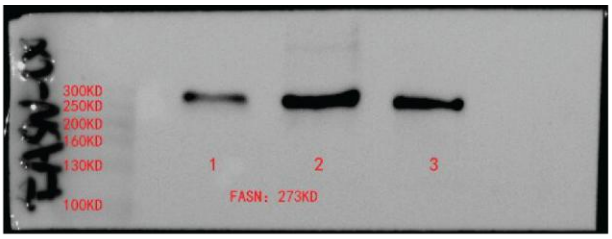
300KD
250KD
200KD
160KD
130KD
100KD
1 2 3
FASN: 273KD
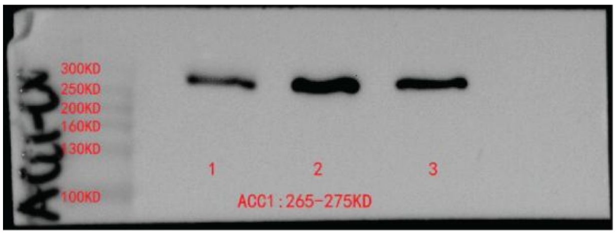
300KD
250KD
200KD
160KD
130KD
100KD
1 2 3
ACC1:265-275KD
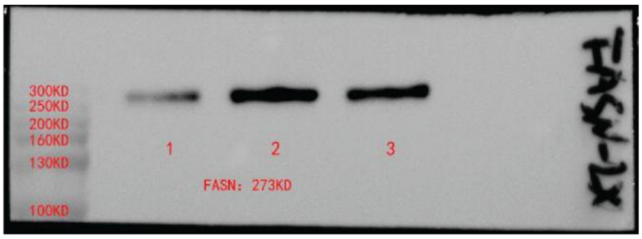
300KD
250KD
200KD
160KD
130KD
100KD
1 2 3
FASN: 273KD
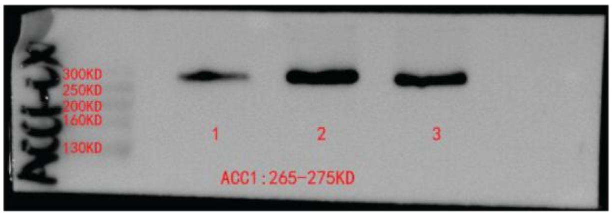
300KD
250KD
200KD
160KD
130KD
1 2 3
ACC1:265-275KD
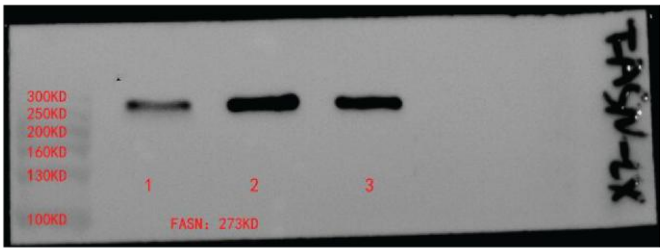
300KD
250KD
200KD
160KD
130KD
100KD
1 2 3
FASN: 273KD
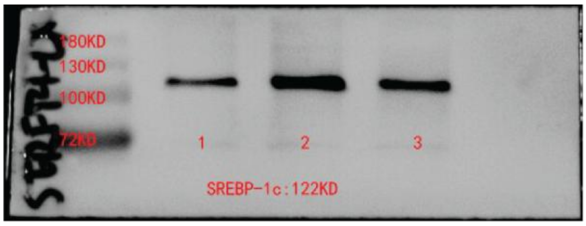
180KD
130KD
100KD
72KD
1 2 3
SREBP-1c:122KD
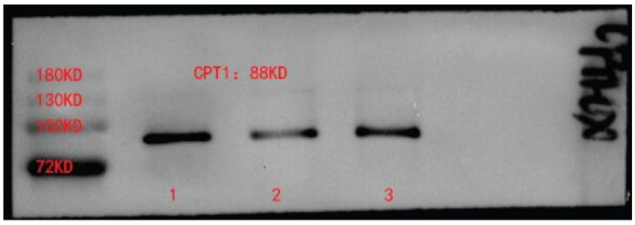
180KD
130KD
100KD
72KD
CPT1: 88KD
1 2 3
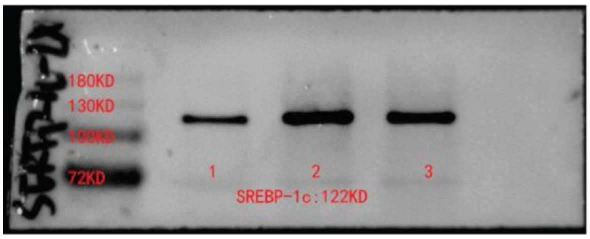
180KD
130KD
100KD
72KD
1 2 3
SREBP-1c:122KD
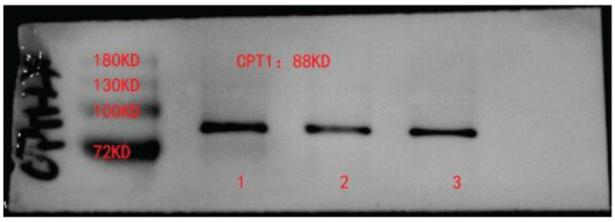
180KD
130KD
100KD
72KD
CPT1: 88KD
1 2 3
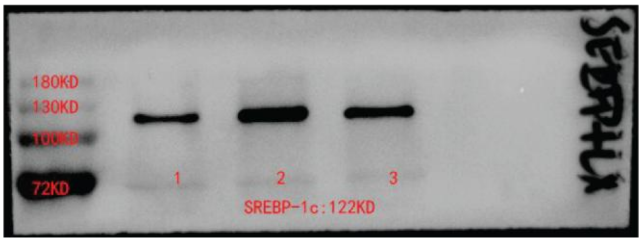
180KD
130KD
100KD
72KD
1 2 3
SREBP-1c:122KD
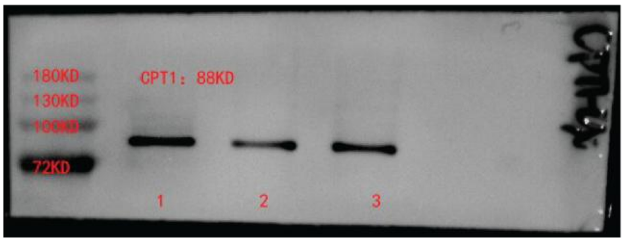
180KD
130KD
100KD
72KD
CPT1: 88KD
1 2 3

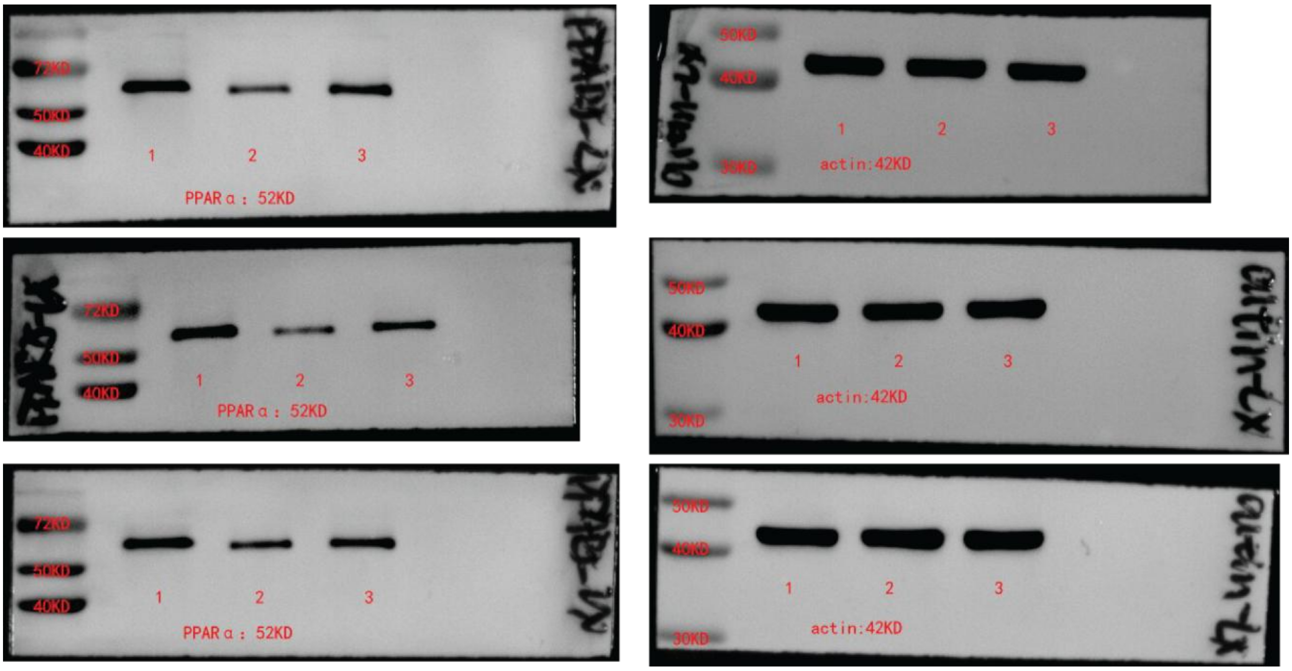

Figure S4 Raw images of Western blot. 1: CND group; 2: HFD group; 3: HFD+ASE group. CND: control normal diet; HFD: high-fat-diet; HFD+ASE: high-fat-diet + aspalathin-rich extract.
